# Microsphere-Based Scaffolds Carrying Opposing Gradients of Chondroitin Sulfate and Tricalcium Phosphate

**DOI:** 10.3389/fbioe.2015.00096

**Published:** 2015-07-01

**Authors:** Vineet Gupta, Neethu Mohan, Cory J. Berkland, Michael S. Detamore

**Affiliations:** ^1^Bioengineering Graduate Program, University of Kansas, Lawrence, KS, USA; ^2^Division of Tissue Engineering and Regeneration Technologies, Biomedical Technology Wing, Sree Chitra Tirunal Institute for Medical Sciences and Technology, Trivandrum, India; ^3^Department of Pharmaceutical Chemistry, University of Kansas, Lawrence, KS, USA; ^4^Department of Chemical and Petroleum Engineering, University of Kansas, Lawrence, KS, USA

**Keywords:** raw materials, chondroitin sulfate, tricalcium phosphate, gradient, microsphere-based scaffolds

## Abstract

Extracellular matrix (ECM) components, such as chondroitin sulfate (CS) and tricalcium phosphate, serve as raw materials, and thus spatial patterning of these raw materials may be leveraged to mimic the smooth transition of physical, chemical, and mechanical properties at the bone-cartilage interface. We hypothesized that encapsulation of opposing gradients of these raw materials in high molecular weight poly(d,l-lactic-co-glycolic acid) (PLGA) microsphere-based scaffolds would enhance differentiation of rat bone marrow–derived stromal cells. The raw material encapsulation altered the microstructure of the microspheres and also influenced the cellular morphology that depended on the type of material encapsulated. Moreover, the mechanical properties of the raw material encapsulating microsphere-based scaffolds initially relied on the composition of the scaffolds and later on were primarily governed by the degradation of the polymer phase and newly synthesized ECM by the seeded cells. Furthermore, raw materials had a mitogenic effect on the seeded cells and led to increased glycosaminoglycan (GAG), collagen, and calcium content. Interestingly, the initial effects of raw material encapsulation on a per-cell basis might have been overshadowed by medium-regulated environment that appeared to favor osteogenesis. However, it is to be noted that *in vivo*, differentiation of the cells would be governed by the surrounding native environment. Thus, the results of this study demonstrated the potential of the raw materials in facilitating neo-tissue synthesis in microsphere-based scaffolds and perhaps in combination with bioactive signals, these raw materials may be able to achieve intricate cell differentiation profiles required for regenerating the osteochondral interface.

## Introduction

A scaffold with opposing gradients of physical and chemical signals at the osteochondral interface may trigger simultaneous bone and cartilage regeneration by having a cooperative effect on tissue regeneration. Our previous studies have shown that 3D microsphere-based gradient scaffolds have the potential to guide the chondro- and osteogenic differentiation of cells in different regions of the scaffolds. Moreover, the gradients in signals have the ability to control patterning of cell phenotype and to secrete tissue-specific extracellular matrix (ECM) components to promote osteochondral interface regeneration (Dormer et al., 2010, 2011, 2012b; Mohan et al., 2011).

Chondroitin sulfate (CS), a glycosaminoglycan (GAG) and a key ECM component of cartilage, when incorporated into 3D scaffolds resulted in increased DNA, GAG, and collagen accumulation by the cultured cells (Uygun et al., 2009; Kim et al., 2014). Moreover, CS also enhanced their chondrogenic gene expression (Chen et al., 2013). Likewise, bioactive ceramic beta-tricalcium phosphate (β-TCP) is widely used in bone tissue engineering because of its excellent oseteoconductivity, cellular adhesion, mechanical properties, and faster degradation rate than other crystalline calcium phosphates. Scaffolds incorporating β-TCP have shown better potential for osteogenic differentiation than the scaffolds without it (Takahashi et al., 2005; Liao et al., 2013; Todo and Arahira, 2013). We have previously demonstrated that encapsulation of raw materials, such as CS and bioactive glass (BG possesses the capability to directly bind to bone), in low molecular weight (around 40–45 kDa) poly(d,l-lactic-co-glycolic acid) (PLGA) microsphere-based scaffolds created a favorable environment for cells to create a tissue-specific ECM. Additionally, evident regional variation in newly synthesized ECM indicated that the raw materials could potentially be used to replace growth factors, thus holding tremendous clinical significance by providing a more streamlined path for regulatory approval and greater financial incentive for translation to the clinic (Mohan et al., 2013).

The low molecular weight PLGA microsphere-based scaffolds are well suited for *in vitro* studies as signal release and cellular response to the encapsulated signals can be conveniently studied in these scaffolds because of rapid degradation of microspheres (Tracy, 1999; Alexis, 2004; Singh et al., 2008; Dormer et al., 2010, 2012a; Mohan et al., 2013). Moreover, these low molecular weight scaffolds can also be used to study tissue regeneration in small animal model *in vivo* studies where skeletal changes occur at a faster rate compared to humans (Pearce et al., 2007; Mohan et al., 2011; Dormer et al., 2012b). In order for a scaffold to be clinically effective and commercially successful, it is imperative that its biodegradation rate matches with the tissue regeneration rate in animal models that closely approximate the human regeneration rate. To begin exploring the clinical implications of our raw material microsphere gradient scaffolds, we need to translate our successes with scaffolds *in vitro* and *in vivo* with small animal models to scaffolds that can be employed in preclinical animal models. The foremost step in that direction will be to study cellular response toward encapsulated factors released from a scaffold system that can be employed in translational animal models (such as sheep, dogs, etc.). Therefore, the objective of this study was to investigate the *in vitro* response of raw material encapsulating microsphere-based scaffolds fabricated with high molecular weight PLGA as a first step to establish the clinical efficacy of these scaffolds. PLGA with an intrinsic viscosity (i.v.) of ~0.7 (M_W_: 106–112 kDa) was chosen for this study due to its relevance in large animal studies (Fonseca et al., 2014), and to correspond to an ongoing sheep study from our group. The polymer formulation used in the study represents a more translational product, inspired by a Coulter Foundation-funded project and input from Food and Drug Administration (FDA) regulatory consultant and business advisors. From this study, we hope to gain an insight into parameters that can have profound implications during *in vivo* experiments.

In this study, we investigated whether the encapsulated raw materials (CS and TCP) in high molecular weight PLGA scaffolds can provide building blocks and facilitate differentiation of the seeded cells simultaneously in the direction of bone- and cartilage-like cells. 3D microsphere-based scaffolds were fabricated using high molecular weight PLGA microspheres encapsulating CS (for cartilage regeneration) and TCP (for bone regeneration) as raw materials. Additionally, scaffolds containing gradient of the raw materials were also fabricated via a gradient technology as previously reported (Singh et al., 2008). The response of rat bone marrow-derived stromal cells (rBMSCs) to the raw materials was evaluated when cultured in a medium consisting of exogenous factors. We hypothesized that encapsulation of raw materials, CS and TCP, in high molecular weight PLGA microsphere-based scaffolds would enhance the differentiation of rBMSCs toward chondrogenic and osteogenic lineages, respectively. Moreover, we anticipate rBMSCs in gradient scaffolds to differentiate simultaneously along an osteochondral route as previously seen in low molecular weight scaffolds encapsulating CS and BG (Mohan et al., 2013).

## Materials and Methods

### Materials

Poly(d,l-lactic-co-glycolic acid) (50:50, lauryl ester end group, *M*_W_ = 106 kDa) with an i.v. of 0.65 dL/g (“PLGA50:50”), and PLGA (75:25, lauryl ester end group, *M*_W_ = 112 kDa) with an i.v. of 0.69 dL/g (“PLGA75:25”) were obtained from Lakeshore Biomaterials (Birmingham, AL, USA). Murine IGF-I was obtained from Peprotech, Inc. (Rocky Hill, NJ, USA). Chondroitin-4-sulfate (lyophilized powder of CS, a sodium salt from bovine trachea) and TCP powder (<200 nm particle) were obtained from Sigma (St. Louis, MO, USA). All other reagents and organic solvents utilized were of cell culture or ACS grade.

### Fabrication of microspheres

Three different types of microspheres were fabricated for the study: (i) PLGA75:25 microspheres (PLGA), (ii) CS–NaHCO_3_ encapsulated PLGA50:50 microspheres (CS), and (iii) TCP-encapsulated PLGA75:25 microspheres (TCP). The rationale for choosing PLGA with two different compositions was to correspond to an on going *in vivo* sheep study from our group. The relatively faster degrading polymer (PLGA50:50) was selected for its ability to release the raw materials quickly in the cartilage region to facilitate chondrogenesis, whereas the slower degrading polymer (PLGA75:25) in the bone region was selected to lend more structural stability to the regenerating tissue. The CS–NaHCO_3_ encapsulated microspheres were fabricated by adding 2% w/v CS and 2% w/v NaHCO_3_ to 16% w/v PLGA50:50 dissolved in dichloromethane (DCM), and the TCP encapsulated microspheres were fabricated by adding 4% w/v TCP to 16% w/v PLGA75:25 dissolved in DCM. Using the PLGA-CS/TCP emulsions, microspheres were fabricated via our previously reported technology (Berkland et al., 2001; Singh et al., 2008; Dormer et al., 2010, 2011, 2012a,b; Mohan et al., 2011, 2013). Briefly, using acoustic excitation produced by an ultrasonic transducer (Branson Ultrasonics, Danbury, CT, USA), regular jet instabilities were created in the polymer stream, thereby creating uniform polymer droplets. An annular carrier non-solvent stream of 0.5% w/v poly (vinyl alcohol) (PVA, 88% hydrolyzed, 25 kDa, Polysciences, Inc., Warrington, PA, USA) in deionized water (DI H_2_O) surrounding the polymer droplets was flowed using a coaxial nozzle that carried the emanated polymer droplets into a beaker containing the non-solvent solution at 0.5% w/v in DI H_2_O, to prevent aggregation of the droplets. The polymer droplets were stirred for 3–4 h to allow for solvent to evaporate, and then filtered and rinsed with DI H_2_O to remove residual PVA, and stored at -20°C. The particles were lyophilized for 48 h before further use.

### Scaffold fabrication

Gradient scaffolds (“GRADIENT” group) were prepared using our previously established technology (Singh et al., 2008; Dormer et al., 2010; Mohan et al., 2011, 2013). In brief, lyophilized microspheres (50–100 mg) of two different types, CS and TCP, were dispersed in DI H_2_O and loaded into two separate syringes. The suspensions were then pumped at opposing flow rates using programmable syringe pumps (PHD 22/2000; Harvard Apparatus, Inc., Holliston, MA, USA) into a cylindrical plastic mold (diameter ~ 4 mm) having a filter at the bottom until a height of about 6 mm was reached. The scaffolds were 3.8–4.0 mm in diameter and around 6 mm in height. The profile for these gradient constructs was linear, where the top one-fourth of the total height comprised of CS microspheres (1.5 mm), then the next one-fourth (1.5 mm) was a linear transition from CS to TCP microspheres, and the remaining half (3 mm) contained only TCP microspheres. The stacked microspheres were then sintered with ethanol-acetone (95:5 v/v) for 55 min. The scaffolds were further lyophilized for 48 h and sterilized with ethylene oxide for 12 h prior to cell seeding experiments. The control PLGA and other homogenous scaffolds, abbreviated as CS and TCP, were fabricated by packing the corresponding microspheres into the same molds, followed by sintering for 55 min, except for PLGA scaffolds (sintered for 45 min). The homogeneous scaffolds had dimensions similar to GRADIENT scaffolds (diameter 3.8–4.0 mm and height 6 mm). A total of four different groups were tested in the study and were named according to the composition of microspheres as: PLGA, CS, TCP, and GRADIENT.

### Cell seeding of scaffolds

Rat bone marrow-derived stromal cells were obtained from the femurs of 10 young male Sprague–Dawley rats (176–200 g, SASCO), following a University of Kansas approved IACUC protocol (175–08) and cultured in medium consisting of α-MEM supplemented with 10% FBS (MSC-Qualified, cat #10437-028) and 1% penicillin–streptomycin (P/S) (all from Invitrogen Life Technologies, Carlsbad, CA, USA). When the cells were 80–90% confluent, they were trypsinized and re-plated at 7,000 cells/cm^2^. Seeding was performed when cells reached P4. Scaffolds were sterilized using ethylene oxide for 12 h, allowed to ventilate overnight after sterilization, and placed in a 24-well plate. Cells (P4) were resuspended in culture medium at a concentration of ~10 million/mL. Eighty microliters of this cell suspension (~750 K cells) were placed directly onto the top of the scaffold, which infiltrated the scaffold via capillary action (Dormer et al., 2012a). Cells were allowed to attach for 1 h after which 2 mL of culture medium was added. After 24 h, the culture medium was replaced by 2 mL of differentiation medium consisting of α-MEM, 1% P/S, 10% FBS, 4 mM β-glycerophosphate (β-GP), 100 nM dexamethasone (DEX) (MP Biomedicals, Santa Ana, CA, USA), 25 mM HEPES buffer (Fisher Scientific, Fairlawn, NJ, USA), and 100 ng/mL murine IGF-I (Peprotech Inc., Rocky Hill, NJ, USA). Every 48 h for 6 weeks, two-thirds of the differentiation medium were replaced with fresh medium.

### Scanning electron microscopy

Scaffolds in culture were fixed in glutaraldehyde followed by dehydration in ethanol. Afterwards, the scaffolds were lyophilized for 48 h prior to imaging. The PLGA, CS, TCP, and GRADIENT acellular (week 0) and cellular (week 1.5) microsphere-based scaffolds were imaged using a LEO 1550 field emission scanning electron microscope at an accelerating voltage of 10 kV.

### Mechanical testing

Unconfined compression tests of the acellular (week 0) and cellular (week 6) microsphere-based scaffolds (*n* = 4–5) were conducted using a uniaxial testing apparatus (Instron Model 5848, Canton, MA, USA) with a 50 N load cell. A custom-made stainless steel bath and compression-plate assembly were mounted in the apparatus (Singh, 2008). Cylindrical scaffold samples were compressed to 80% strain at a strain rate of 1%/s under phosphate-buffered saline [PBS: 0.138M sodium chloride, 0.0027M potassium chloride] at 37°C. Among all possible testing modalities, compression at a 1%/s strain rate provides the most valuable information in terms of achieving high strain levels to view the entire stress-strain profile, which cyclic testing and stress relaxation/creep testing do not provide, and moreover a reproducible elastic modulus can be obtained without preconditioning as we have done in the past (Detamore and Athanasiou, 2003). Compressive moduli of elasticity were calculated from the initial linear regions, ~5% strain, of the stress–strain curves as described previously (Singh et al., 2008; Dormer et al., 2010, 2012a; Mohan et al., 2013).

### Porosity measurement

We have previously demonstrated a close match between theoretical porosities and porosities measured by porosimetry and microCT (Singh et al., 2008; Jeon et al., 2013). Therefore, a fluid saturation method was used in this study to calculate the porosities of the scaffolds:

$$\begin{array}{l} V_{B}=4m÷\pi d^{2}h,\\ W_{\mathrm{Water}}=W_{W}-W_{D},\\ V_{P}=W_{\mathrm{Water}}÷\rho_{\mathrm{Water}},\\ \mathrm{Porosity}\left(\varphi \right)\left(\%\right)=\left(V_{P}÷V_{B}\right)\times 100 \end{array}$$

where *V*_B_, *m*, *d*, *h*, *W*_W_, *W*_D_, and *V*_P_ are the bulk volume, mass, diameter, height, wet weight, dry weight, and pore volume of the scaffolds, respectively. *W*_Water_ and ρ_Water_ are the weight and density of water. Briefly, wet and dry weights of scaffolds were recorded after fabrication and porosities were determined by the above-described method.

### Biochemical analyses

Engineered constructs (*n* = 5) were analyzed for matrix production at 0, 3, and 6 weeks. The samples were digested in two different types of digestion solution: (i) Papain solution for DNA, GAG, and hydroxyproline (HYP) content analyses, and (ii) Triton-X solution for calcium content and alkaline phosphatase (ALP) activity analyses. The papain digestion solution consisted of 125 mg/mL papain (from papaya latex), 5 mM *N*-acetyl cysteine, 5 mM ethylenediaminetetraacetic acid, and 100 mM potassium phosphate buffer (20 mM monobasic potassium phosphate, 79 mM dibasic potassium phosphate) (all reagents from Sigma Aldrich) in DI H_2_O. Engineered constructs were removed from culture in a sterile manner, placed in microcentrifuge tubes, homogenized with the papain solution (1 mL), and allowed to digest overnight in a 60°C water bath. The digested scaffolds were then centrifuged at 10,000 rpm for 5 min to pellet fragments of polymer and other impurities and stored at -20°C. Later, the supernatant was used to determine DNA, GAG, and HYP contents using the Picogreen (Molecular Probes, Eugene, OR, USA), dimethylmethylene blue (DMMB) (Biocolor, Newtownabbey, Northern Ireland), and HYP (cat #MAK008, Sigma Aldrich, St. Louis, MO, USA) assays, respectively. For calcium and ALP analyses, constructs were digested in 0.05% Triton X-100 and the supernatants were placed in the -20°C before the analyses. Calcium content was assessed using a QuantiChromTM Calcium Assay Kit (DICA-500; QuantiChrom, Hayward, CA, USA). ALP activity was estimated by determining liberated p-nitrophenol (p-NITRO) rate (concentration/μg DNA per minute) as described elsewhere (Boyan et al., 1989). In the cases of GAG and calcium content, the values of acellular controls for CS and TCP groups (listed in Tables S1 and S2 in Supplementary Material), respectively, were subtracted from the corresponding values of the cellular scaffolds at each time point in an effort to distinguish the bioactivity provided by the CS and TCP from the amounts retained in the scaffolds.

### Gene expression analyses

Reverse transcriptase quantitative polymerase chain reaction (RT-qPCR) was performed for gene expression analyses in microsphere-based constructs (*n* = 3–5) at weeks 0, 1.5, 3, and 6. Certain groups at certain time points (indicated in Section “Results”) had insufficient sample size (*n* < 3) because some of the samples were lost during processing. In preparation for RT-qPCR, samples were first homogenized in 1 mL of Trizol reagent (Invitrogen), and the RNA was isolated according to the manufacturer’s guidelines. Isolated RNA was cleaned using an RNeasy spin column method (Qiagen, Valencia, CA, USA) and converted to complementary DNA using a TaqMan High Capacity kit (Applied Biosystems, Foster City, CA, USA) in an Eppendorf Realplex Mastercycler. TaqMan Gene expression assays from Applied Biosystems for appropriate genes (Table 1) were run in the Eppendorf system. A 2^-ΔΔCt^ method was used to evaluate the relative level of expression for each target gene. For quantification, the PLGA constructs at week 0 were designated as the calibrator group and GAPDH expression as the endogenous control.

**Table 1:** **Genes used for RT-qPCR analysis**.

Gene	Symbol	TaqMan assay ID
Glyceraldehyde 3-phosphate dehydrogenase	GAPDH	Rn01775763_g1
SRY (sex determining region Y)-box 9	SOX9	Rn01751069_mH
Collagen type II	COL2A1	Rn01751069_mH
Aggrecan	ACAN	Rn00573424_m1
Collagen type I	COL1A1	Rn01463848_m1
Runt-related transcription factor 2	RUNX2	Rn01512298_m1
Bone gamma-carboxyglutamate protein	BGLAP	Rn00566386_g1
Secreted phosphoprotein 1	SPP1	Rn01449972_m1
Integrin-binding sialoprotein	IBSP	Rn00561414_m1

### Statistical analyses

SPSS 21.0 (IBM, Armonk, NY, USA) was used for constructing standard box plots for outlier elimination. For statistical inference in Sections “Mechanical Testing” and “Porosity Measurement”, a single factor analysis of variance (ANOVA) was performed with SPSS, followed by a Tukey’s honestly significant difference *post hoc* test when significance was detected below the *p* = 0.05 value. In Sections “Biochemical Analyses” and “Gene Expression Analyses”, the statistical inference was performed using a two-factor ANOVA followed by a Tukey’s honestly significant difference *post hoc* test when significance was detected below the *p* = 0.05 value. The model included the two factors (scaffold type and time) and the possible interactions between them. All quantitative results (numerical values and representative diagrams) are expressed as the average ± SD.

## Results

### Scanning electron microscopy

**Figure 1:**
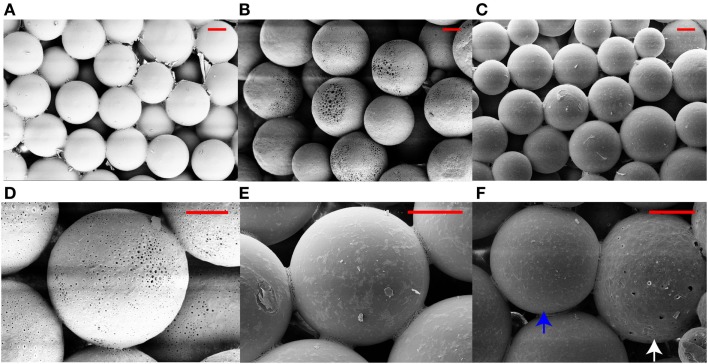
**Scanning electron micrographs of acellular microsphere-based scaffolds**. The images reveal the distinct morphological features of the microspheres in different scaffold groups: **(A)** PLGA, **(B,D)** CS, **(C,E)** TCP, and **(F)** GRADIENT at the CS (white arrow)-TCP (blue arrow) transition region. Scale bar: 100 μm.

**Figure 2:**
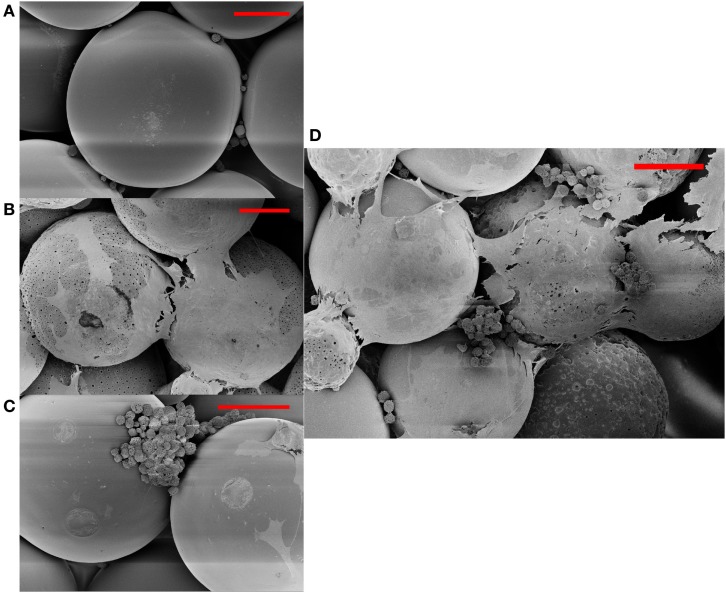
**Cellular morphology of rBMSCs at day 10 (week 1.5) on different scaffold groups: (A) PLGA, (B) CS, (C) TCP, and (D) GRADIENT as depicted by the scanning electron micrographs**. Scale bar: 100 μm.

Figures 1 and 2 represent the scanning electron micrographs of all four types of scaffolds. Figure 1 demonstrates that the fabricated microspheres were uniform in size (Figure S1 in Supplementary Material) and also illustrates the overall porous nature of microsphere-based scaffolds with interconnected pores. Additionally, it highlights the differences in microsphere morphology among the various scaffold groups. The microspheres in PLGA-only scaffolds (Figure 1A) were smooth with surface film layers being formed as a result of plasticization of PLGA with ethanol-acetone (Singh et al., 2008). The microspheres in CS scaffolds (Figures 1B,D) had minute pores on their surface while the microspheres in TCP scaffolds had a rougher appearance (Figures 1C,E) than microspheres in the PLGA-only group. The GRADIENT scaffold image (Figure 1F) shows fusion between porous (CS) and rough (TCP) microspheres at the transition region of the scaffold. Apart from the differences in microsphere structure, variations were also observed in the cellular morphology of the cell-seeded constructs (Figure 2). At Day 10 (week 1.5), very few cells were observed in the PLGA-only scaffolds residing in pores between the adjacent microspheres, and these cells possessed a rounded morphology (Figure 2A). By contrast, a far greater number of cells could be seen in the other three groups with differences appearing in the cellular morphologies. Cells covered the surface of the microspheres almost completely in the CS scaffolds and appeared to be flat with cell–cell connections being evident at the sintering junctions between the adjacent microspheres (Figure 2B). Cells in the TCP scaffolds had a round appearance, and were clustered around the microsphere sintering junctions (Figure 2C). Both cell types with round (in clusters) and flat morphologies were present in the GRADIENT group (Figure 2D). However, no apparent morphological differences were observed in cells from distinct regions of the GRADIENT scaffold.

### Mechanical testing

**Figure 3:**
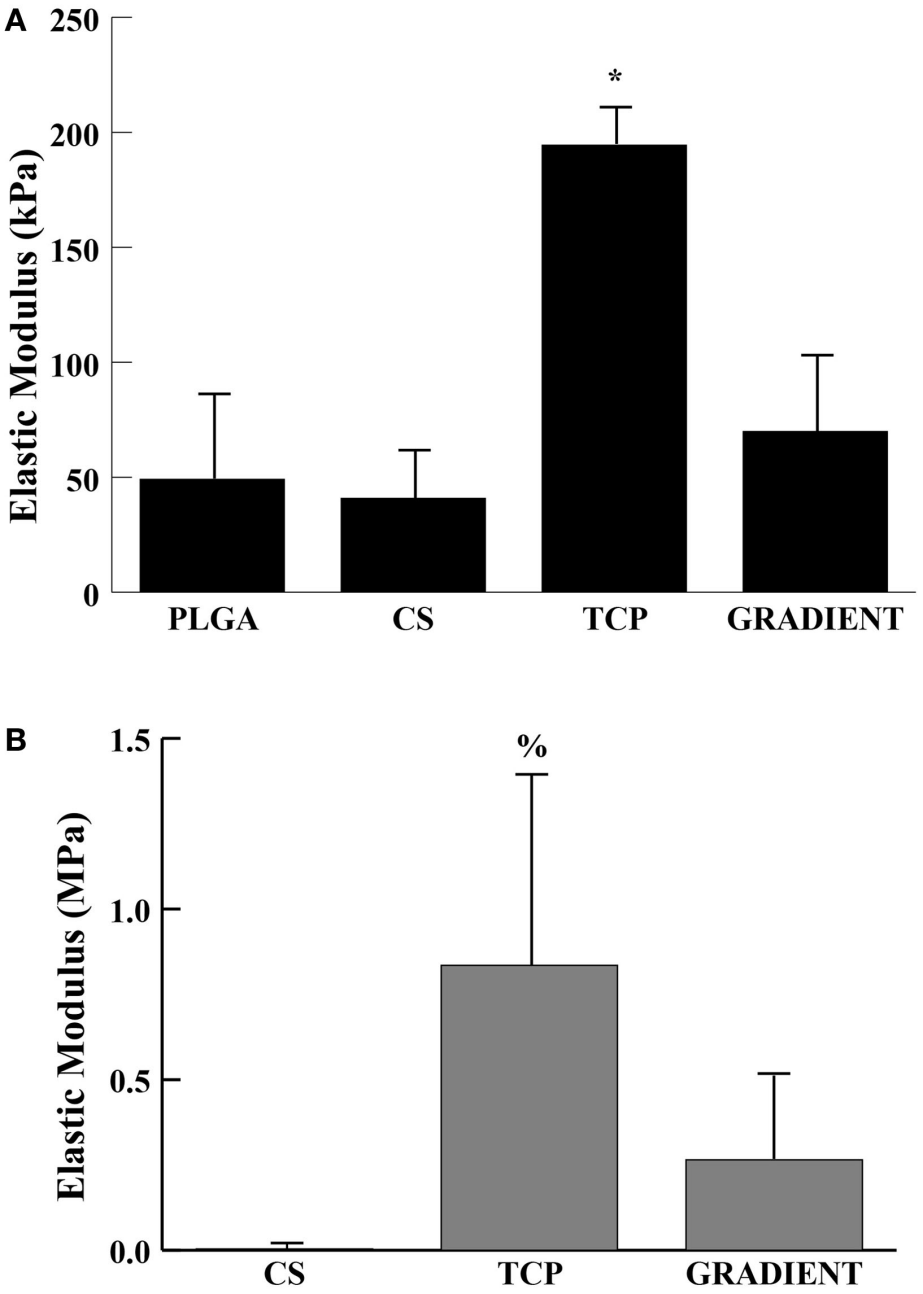
**Elastic modulus**. **(A)** Acellular constructs at week 0. **(B)** Cellular constructs at week 6. All values are expressed as the average ± SD (*n* = 3–5), *p* < 0.05 *Statistically significant change over the other three groups, ^%^statistically significant change over the CS group. PLGA constructs at week 6 are not shown in the figure.

Tricalcium phosphate acellular scaffolds had an average elastic modulus of 194 ± 16 kPa at week 0 that was 4- (*p* < 0.05), 4.8- (*p* < 0.05), and 2.8-fold (*p* < 0.05) higher than the moduli of PLGA, CS, and GRADIENT scaffolds, respectively (Figure 3A). Additionally, among the cell-seeded scaffolds, TCP constructs at week 6 had an average modulus of 0.84 ± 0.55 MPa that was 208.8-fold (*p* < 0.05) higher than the modulus of the CS group (Figure 3B). Surprisingly, it was observed that the PLGA constructs at week 6 had an average modulus of 11.4 ± 6.6 MPa (not shown in the figure) that was orders of magnitude higher than the moduli of the other three groups at that time. No significant differences were observed between the elastic moduli of CS and GRADIENT groups at week 6.

### Porosity measurement

The average porosity of CS group was 49.6 ± 4.4% that was 2.4-fold (*p* < 0.05) higher than the porosity of the PLGA group (Table 2). Moreover, the porosities of the scaffolds in the CS group were also statistically significantly higher than the porosities of their counterparts in the TCP and GRADIENT groups. No significant differences in porosities were observed among any other groups.

**Table 2:** **Average porosities of different scaffold groups**.

Group	Average Porosity (%)
PLGA	21.0 ± 6.8
CS	49.6 ± 4.4*
TCP	21.6 ± 6.8
GRADIENT	18.4 ± 4.6

*Statistically significantly higher than the other three groups.

### Biochemical analysis

#### DNA Content

**Figure 4:**
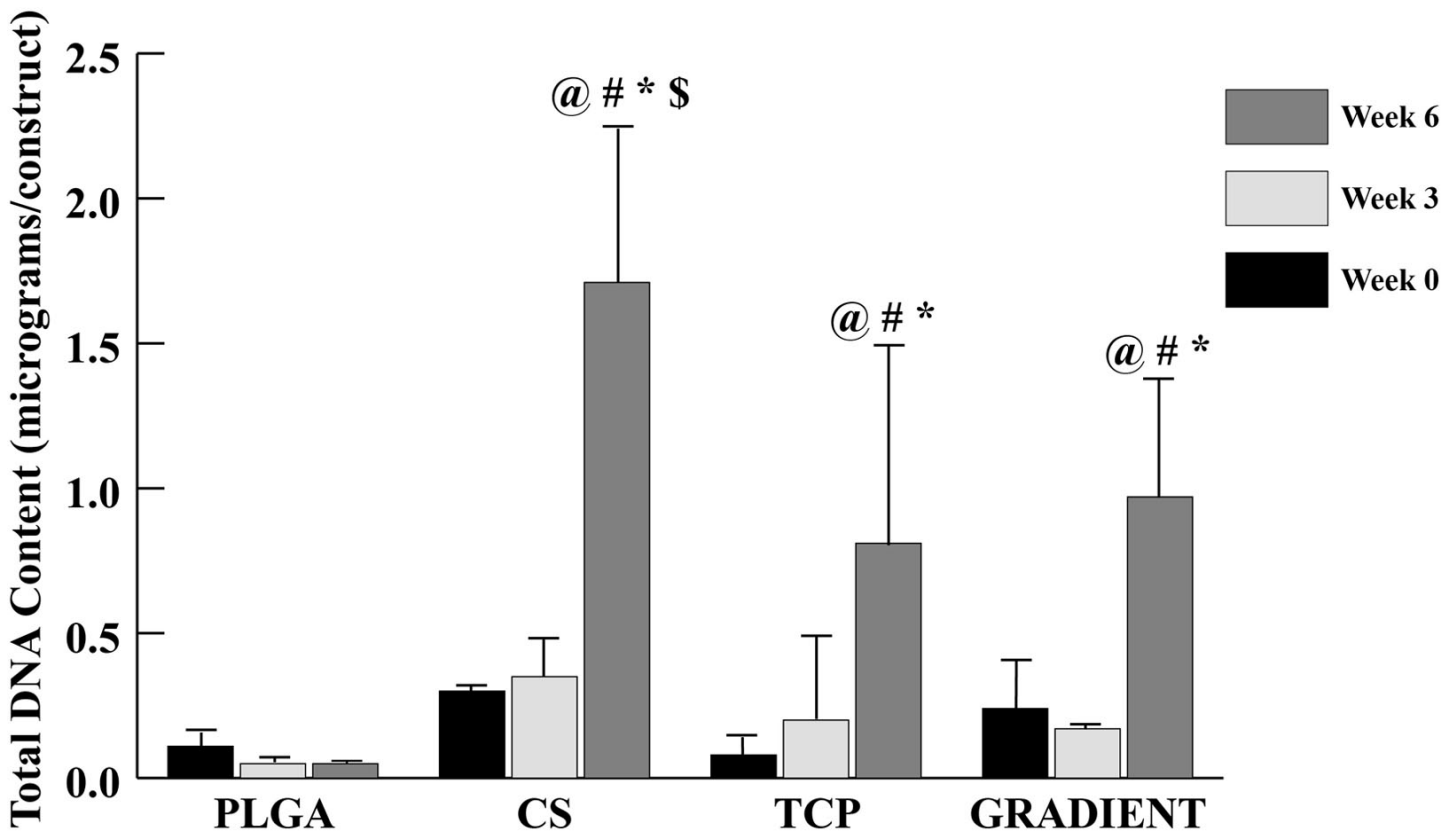
**Total DNA content as measured in the microsphere-based scaffolds over weeks 0, 3, and 6**. All values are expressed as the average ± SD (*n* = 3–5), *p* < 0.05. ^@^Statistically significant change over week 0 value, ^#^statistically significant change over its value at previous time point, *statistically significant change over the PLGA group at same time point, and ^$^statistically significant change over the TCP and GRADIENT groups at same time point.

The DNA content results (Figure 4) revealed no significant differences in the amount of DNA present in the four distinct types of scaffolds at weeks 0 and 3. At week 6, the DNA content in CS scaffolds was 31.7-fold (*p* < 0.05) higher than the DNA content in the PLGA group. The TCP and GRADIENT groups also outperformed the PLGA control at week 6, with 15- (*p* < 0.05) and 18-fold (*p* < 0.05) higher DNA contents, respectively. Moreover, the DNA content in the CS group at week 6 was statistically significantly higher than the DNA contents in the TCP and GRADIENT groups at that time. Additionally, the CS, TCP, and GRADIENT groups were observed to have statistically significantly higher DNA content at week 6 than their corresponding values at weeks 0 and 3; however, no significant differences in the DNA content over time were observed in the PLGA group.

#### GAG Content

**Figure 5:**
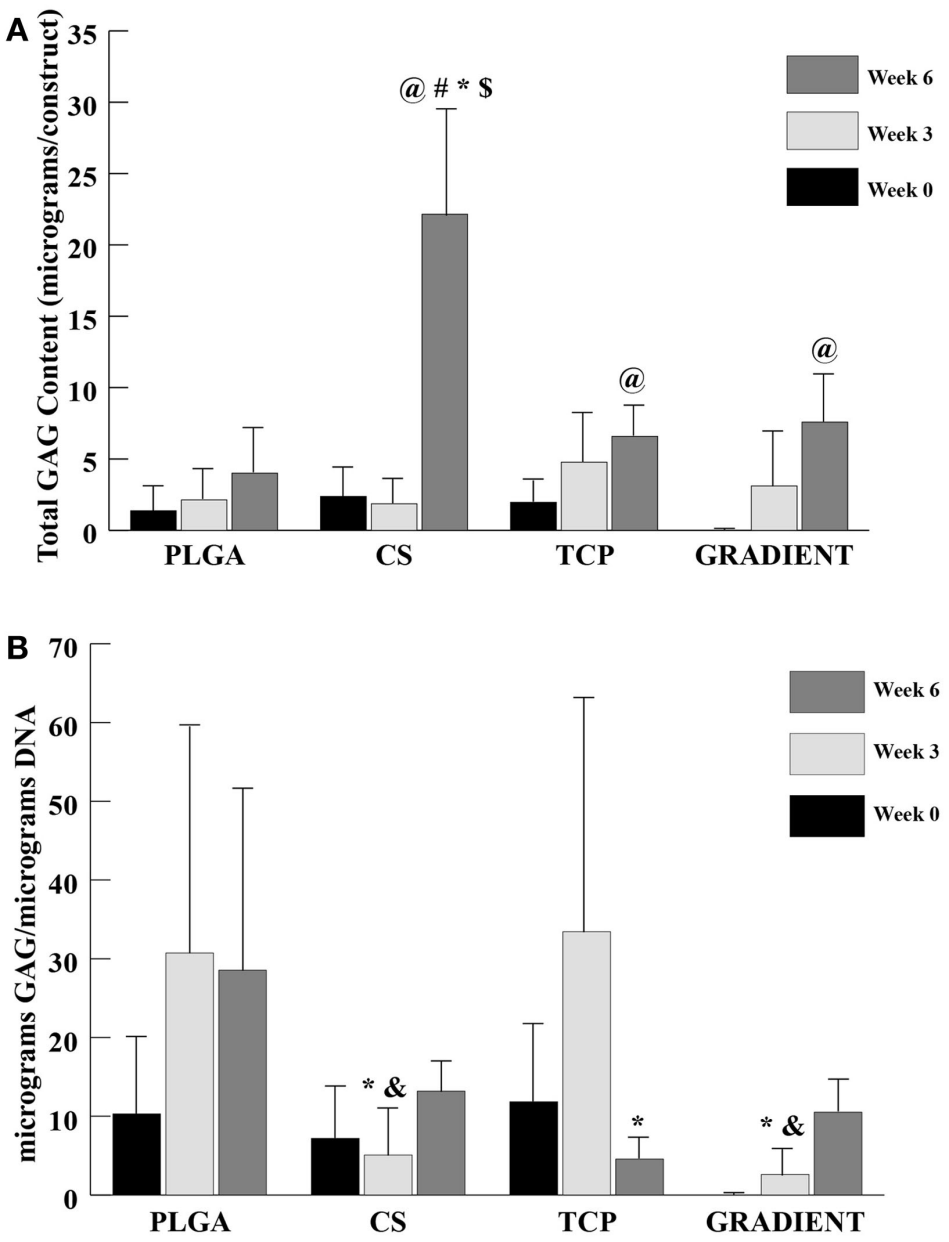
**GAG content as measured in the microsphere-based scaffolds over weeks 0, 3, and 6**. GAG content in the acellular constructs (CS and GRADIENT groups) was subtracted from the GAG content in the corresponding cellular constructs. **(A)** Total GAG content in micrograms per construct. **(B)** Normalized GAG content in micrograms per micrograms DNA. All values are expressed as the average ± SD (*n* = 3–5), *p* < 0.05. ^@^Statistically significant change over week 0 value, ^#^statistically significant change over its value at previous time point, *statistically significant change over the PLGA group at same time point, ^$^statistically significant change over the TCP and GRADIENT groups at same time point, and ^&^statistically significant change over the TCP group at same time point.

A trend similar to DNA content was observed in the GAG content (Figure 5A), where no significant differences appeared among groups at weeks 0 and 3. At week 6, the net GAG content of the CS scaffolds was 5.5-fold (*p* < 0.05) higher than the GAG content of the PLGA group. Moreover, the GAG content in the CS group at week 6 was also statistically significantly higher than the GAG contents in the TCP and GRADIENT groups. No significant differences in the GAG content were observed among the other three groups week 6, meaning that the CS group was the only group to statistically significantly outperform the PLGA control at that time. The GAG content in the CS scaffolds (22.2 ± 7.5 μg) at week 6 was found to be statistically significantly higher than its corresponding values at weeks 0 and 3. The TCP and the GRADIENT groups had significantly higher GAG content at week 6 than their respective values at week 0. Furthermore, the PLGA and TCP groups at week 3 had significantly higher GAG content when it was normalized to the DNA content than the normalized GAG content of the CS and GRADIENT groups at that time (Figure 5B). However, at week 6, only the TCP group statistically significantly differed from the PLGA group in the normalized GAG content. It must be noted that the values of GAG content obtained from the biochemical analysis represent both the GAGs present in the ECM secreted by the cells and the CS released by the scaffold and then entrapped within the ECM. The values *do not* represent the CS left entrapped within the polymer matrix, as the GAG content of acellular controls was subtracted at each time point.

#### HYP Content

**Figure 6:**
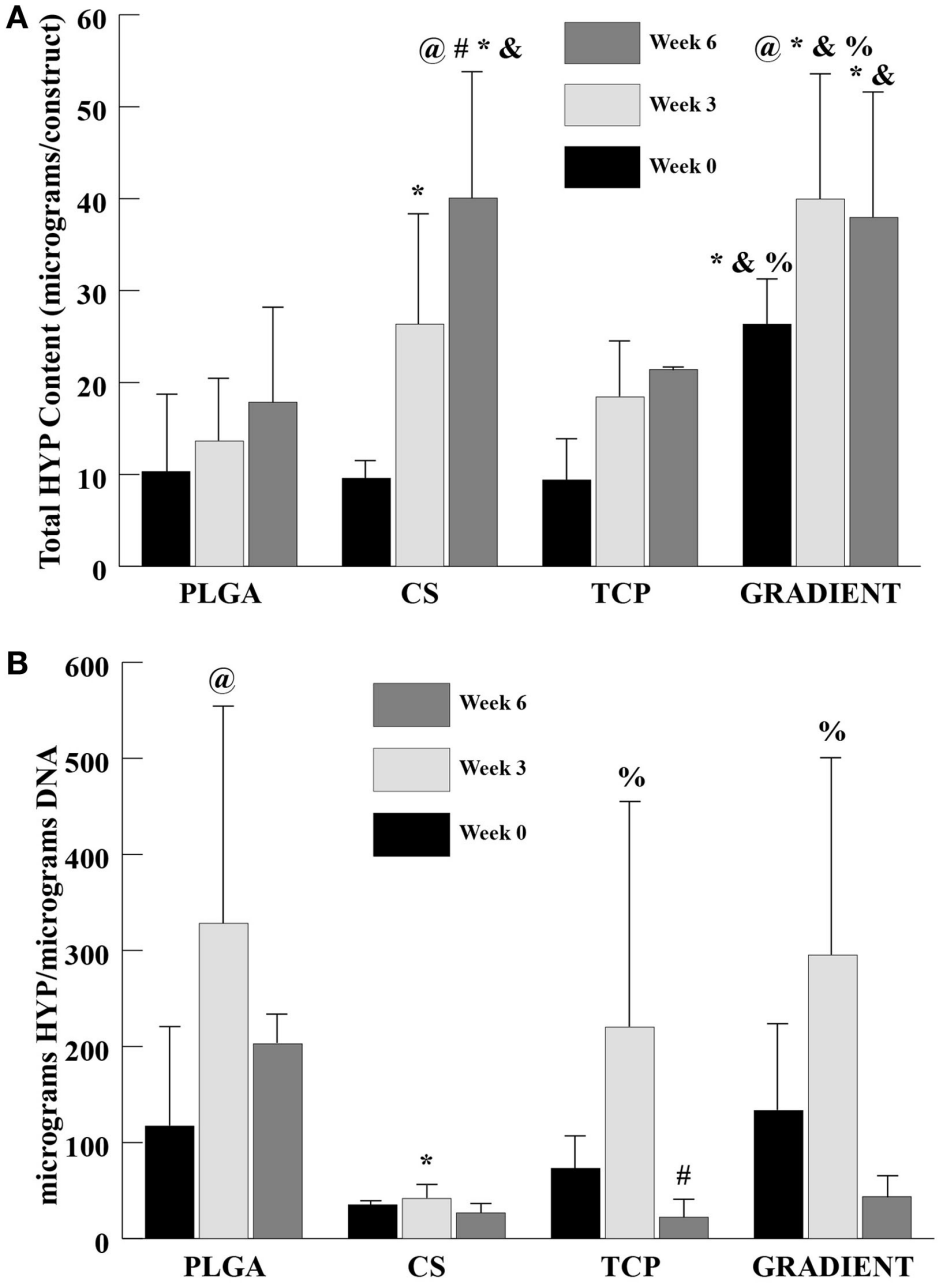
**HYP content as measured in the microsphere-based scaffolds over weeks 0, 3, and 6**. **(A)** Total HYP content in micrograms per construct. **(B)** Normalized HYP content in micrograms per micrograms DNA. All values are expressed as the average ± SD (*n* = 3–5), *p* < 0.05. ^@^Statistically significant change over week 0 value, ^#^statistically significant change over its value at previous time point, *statistically significant change over the PLGA group at same time point, ^&^statistically significant change over the TCP group at same time point, and ^%^statistically significant change over the CS group at same time point.

At week 0, only the GRADIENT group outperformed the PLGA group in HYP content with 2.6-fold (*p* < 0.05) higher HYP content (Figure 6A). Moreover, the HYP content in the GRADIENT group at week 0 was statistically significantly higher than the HYP contents in the CS and TCP groups. Week 3 HYP content results showed that the CS and GRADIENT groups had 1.9- (*p* < 0.05) and 2.9-fold higher HYP content than the PLGA group, respectively. Also, the GRADIENT group at week 3 had statistically significantly higher HYP content than the CS and TCP groups. At week 6, both the CS and the GRADIENT groups outperformed the PLGA control, with HYP contents that were 2.2- (*p* < 0.05) and 2.1-fold (*p* < 0.05) higher, respectively. Additionally, the CS and GRADIENT groups had statistically significant higher HYP contents than the TCP group at week 6. The CS and GRADIENT groups were the only groups that showed statistically significant increases in HYP content over time. The HYP content in the CS group at week 6 was significantly higher than its corresponding values at weeks 0 and 3, whereas the HYP content in the GRADIENT group at week 3 was significantly higher than its HYP content at week 0. In the normalized HYP (per DNA) content, the PLGA, CS, and the GRADIENT groups were statistically significantly higher than the CS group at week 3 (Figure 6B), with no significant differences occurring in the normalized HYP content among the PLGA, CS, and GRADIENT groups. The PLGA and TCP groups at week 3 had statistically significantly higher normalized HYP content than their values at week 0 and 6, respectively. No significant differences were observed in the CS and GRADIENT groups over time in the normalized HYP content.

#### Calcium Content

**Figure 7:**
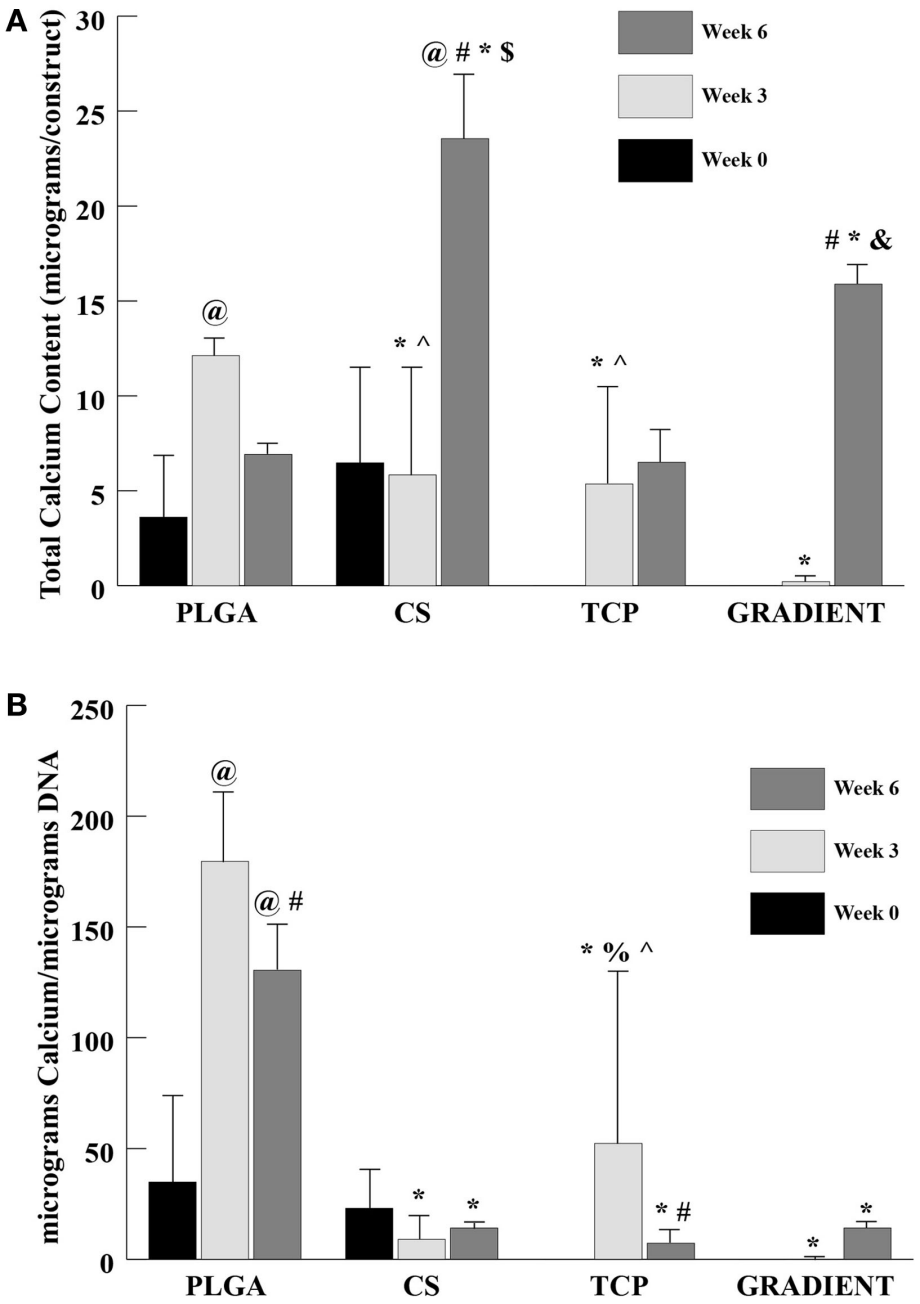
**Calcium content as measured in the microsphere-based scaffolds over weeks 0, 3, and 6**. Calcium content in the acellular constructs (TCP and GRADIENT groups) was subtracted from the calcium content in the corresponding cellular constructs. The calcium contents of the TCP and GRADIENT constructs at week 0 are not reported because of insufficient sample size (*n* < 3). **(A)** Total calcium content in micrograms per construct. **(B)** Normalized calcium content in micrograms per micrograms DNA. All values are expressed as the average ± SD (*n* = 3–5), *p* < 0.05. ^@^Statistically significant change over week 0 value, ^#^statistically significant change over its value at previous time point, *statistically significant change over the PLGA group at same time point, ^$^statistically significant change over the TCP and GRADIENT groups at same time point, ^&^statistically significant change over the TCP group at same time point, ^^^statistically significant change over the GRADIENT group at same time point, and ^%^statistically significant change over the CS group at same time point.

The calcium content analysis revealed no significant differences between the PLGA and CS groups at week 0 (Figure 7A). The calcium contents of TCP and GRADIENT scaffolds at week 0 are not reported because of insufficient sample size (*n* < 3), as some of the samples were lost during processing. At week 3, the calcium content in the PLGA group was statistically significantly greater than the calcium contents in the CS, TCP, and GRADIENT groups. Also, the calcium contents in the CS and TCP groups at week 3 were statistically significantly higher than the calcium content in the GRADIENT group. At week 6, the calcium contents in the CS and GRADIENT groups were 3.4- (*p* < 0.05) and 2.3-fold (*p* < 0.05) greater than the calcium content of the PLGA group. Moreover, the CS group calcium content at week 6 was observed to be statistically significantly higher than the calcium contents of the TCP and GRADIENT groups, and the GRADIENT group was found to be significantly higher than the TCP group in calcium content at week 6. No significant differences were observed in the calcium contents of the PLGA and TCP groups at that time, meaning that only the CS and GRADIENT groups outperformed the PLGA control in calcium content at week 6. The calcium content of the PLGA group increased statistically significantly at week 3 from its week 0 value, followed by a decrease at week 6 that was not statistically significant. The CS group had significantly higher calcium content at week 6 than at weeks 0 and 3. In addition, the GRADIENT group had significantly more calcium at week 6 than at week 3. No significant differences in calcium content of the TCP group were observed over time. The normalized calcium content (Figure 7B) of PLGA scaffolds at week 3 was statistically significantly higher than the normalized calcium contents in the CS, TCP, and GRADIENT groups. Additionally, the normalized calcium content in the TCP group at week 3 was statistically significantly higher than the normalized calcium contents of the CS and GRADIENT groups. Furthermore, at week 6, the PLGA group’s normalized calcium content was significantly higher than the normalized calcium content in the CS, TCP, and GRADIENT groups. The normalized calcium contents in the PLGA group at weeks 3 and 6 were statistically significantly higher than its corresponding value at week 0. However, the normalized calcium contents in the PLGA and TCP groups at week 6 were statistically significantly lower than their corresponding values at week 3. Again, it is to be emphasized that the values of calcium content are intended to represent the calcium present in the ECM secreted by the cells, and the calcium released from the microspheres and retained by the construct, and not the calcium still entrapped within the polymeric matrix.

#### ALP Activity

**Figure 8:**
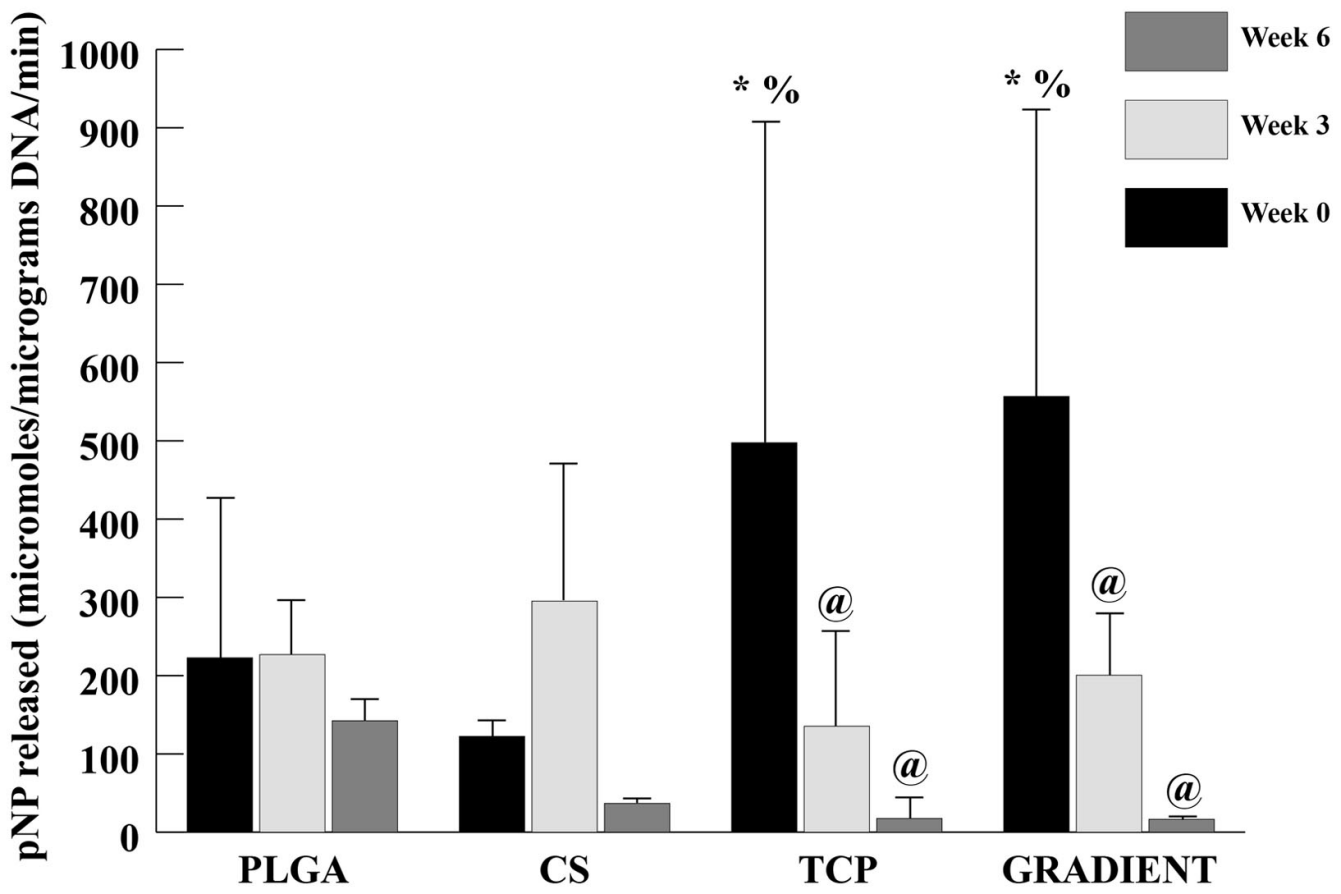
**ALP activity in micromolar pNP released per micrograms DNA per minute**. All values are expressed as the average ± SD (*n* = 3–5), *p* < 0.05. ^@^Statistically significant change over week 0 value, *statistically significant change over the PLGA group at same time point, and ^%^statistically significant change over the CS group at same time point.

At week 0, the ALP activities in the TCP and GRADIENT groups were 2.2- (*p* < 0.05) and 2.5-fold (*p* < 0.05) higher than the ALP activity in the PLGA group (Figure 8). Moreover, the ALP activities in the TCP and GRADIENT groups at week 0 were statistically significantly higher than the ALP activity in the CS group. No significant differences were observed in the ALP activities of PLGA and CS groups at week 0, meaning that only the TCP and GRADIENT groups outperformed the PLGA control in ALP activity at that time point. No significant differences in ALP activity were observed over time in the PLGA and CS groups. However, it was observed that the ALP activities of the TCP and GRADIENT groups at week 0 were statistically significantly higher than their corresponding values at weeks 3 and 6.

### Gene expression

#### SOX9 and COL2A1

**Figure 9:**
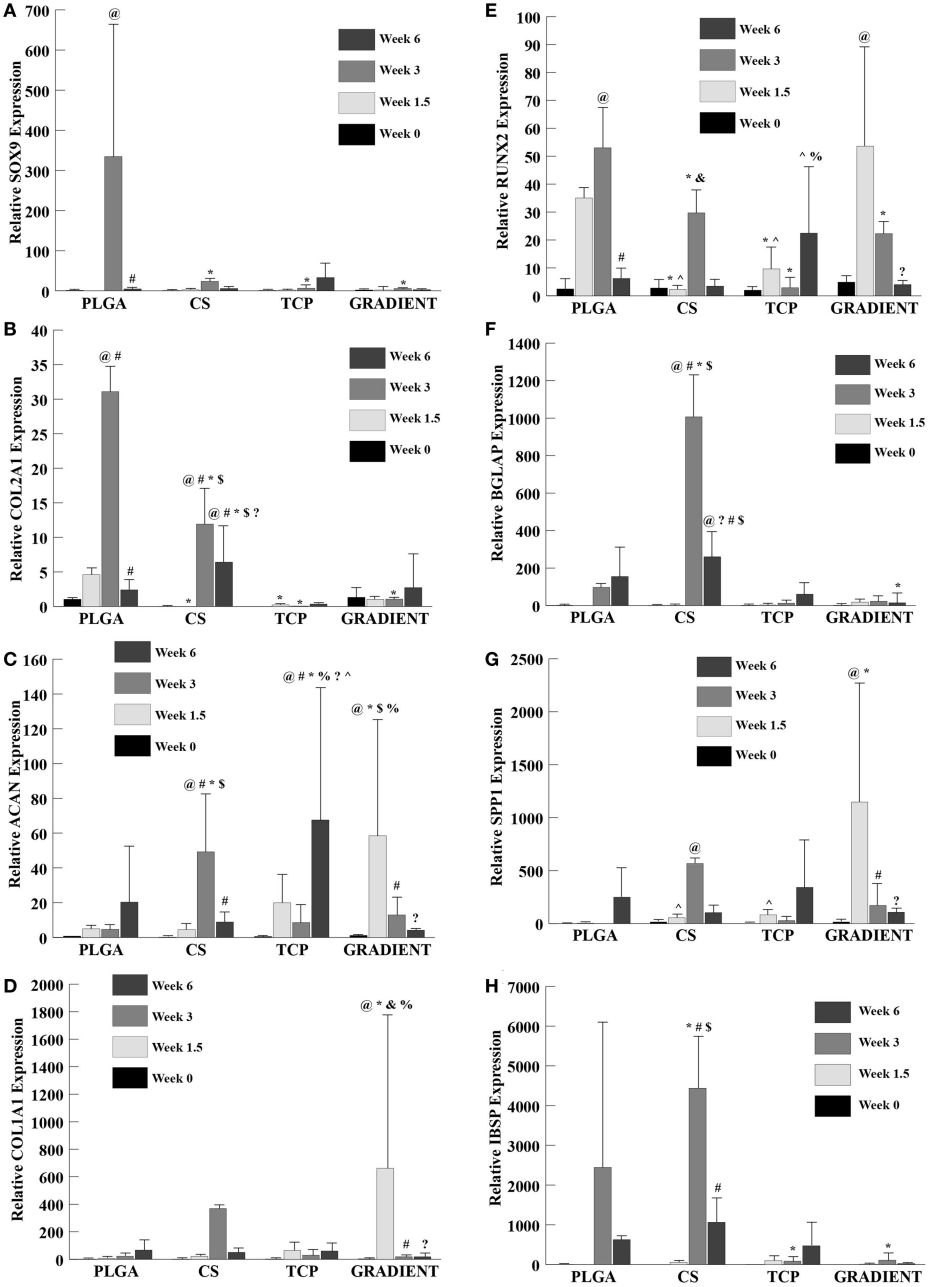
**Relative gene expression**. All values are expressed as the average ± SD (*n* = 3–5). **(A)** SOX9 expression: the PLGA group at week 1.5 is not reported because of insufficient sample size (*n* < 3). **(B)** COL2A1 expression: the TCP group at week 0 is not reported because of insufficient sample size (*n* < 3). **(C)** ACAN expression. **(D)** COL1A1 expression. **(E)** RUNX2 expression. **(F)** BGLAP expression: the PLGA value at week 1.5 is not reported because of insufficient sample size (*n* < 3). **(G)** SPP1 expression. **(H)** IBSP expression: the PLGA group at week 1.5 is not reported because of insufficient sample size (*n* < 3), *p* < 0.05. ^@^Statistically significant change over week 0 value, ^#^statistically significant change over its value at previous time point, *statistically significant change over the PLGA group at same time point, ^$^statistically significant change over the TCP and GRADIENT groups at same time point, ^?^statistically significant change over week 1.5 value, ^%^statistically significant change over the CS group at same time point, ^&^statistically significant change over the TCP group at same time point, and ^^^statistically significant change over the GRADIENT group at same time point.

Relative SOX9 expression (Figure 9A) showed no significant differences among groups at week 0 and also no significant differences among the CS, TCP, and GRADIENT groups at week 1.5. The SOX9 expression for PLGA group is not reported at week 1.5 due to insufficient sample size (*n* < 3 as some of the samples were lost during processing). The SOX9 expression for the PLGA group at week 3 was statistically significantly higher than the SOX9 expression of the CS, TCP, and GRADIENT groups. No significant differences among groups were observed in the SOX9 expression at week 6. The PLGA group was found to have statistically significantly higher SOX9 expression at week 3 than at weeks 0 and 6. No significant differences over time were observed in SOX9 expression within any of the other three groups.

The COL2A1 (collagen II) expression (Figure 9B) in the PLGA group followed a trend similar to SOX9 expression. No significant differences were observed in COL2A1 expression among the PLGA, CS, and GRADIENT groups at week 0 (the TCP group collagen II expression at week 0 is not reported due to insufficient sample size). The COL2A1 expression of the PLGA group at week 1.5 was statistically significantly higher than the COL2A1 expression of the CS and TCP groups. Additionally, the PLGA group had statistically significantly higher COL2A1 expression than the CS, TCP, and the GRADIENT groups at week 3. The CS group at week 3 had significantly higher COL2A1 expression than the TCP and GRADIENT groups. No significant differences in COL2A1 expression between the other two groups were observed at week 3. The CS group at week 6 outperformed the PLGA group in COL2A1 expression with 2.7-fold (*p* < 0.05) higher expression. Moreover, the CS group was statistically significantly higher in COL2A1 expression than the TCP and GRADIENT groups. No significant differences were observed in COL2A1 expression among the other three groups at that time, meaning that only the CS group outperformed the PLGA control group in COL2A1 expression at week 6. The COL2A1 expression in the PLGA and CS groups peaked at week 3 with statistically significant higher expression at week 3 than their respective values at earlier time points of weeks 0 and 1.5; however, the expression values in these groups decreased significantly at week 6 compared to their week 3 COL2A1 expression values. No significant differences over time were observed within the other two groups.

#### ACAN and COL1A1

No significant differences among groups were observed in the ACAN (aggrecan) expression at week 0 (Figure 9C). At week 1.5, the ACAN expression in the GRADIENT group was 11.8-fold (*p* < 0.05) higher than the PLGA group. Moreover, the ACAN expression in the GRADIENT group at week 1.5 was statistically significantly higher than the CS and TCP groups. No significant differences among other three groups were observed in the ACAN expression at that time, meaning that only the GRADIENT group outperformed the PLGA control group in ACAN expression at week 1.5. The ACAN expression of the CS group at week 3 was 10.5-fold (*p* < 0.05) higher than the PLGA group. In addition, the ACAN expression in the CS group at week 1.5 was significantly higher than the expression levels in the TCP and GRADIENT groups. Only the CS group outperformed the PLGA group in ACAN expression at week 3, as no significant differences were observed in ACAN expression among the other three groups. At week 6, the TCP group alone outperformed the PLGA group in ACAN expression with a 3.3-fold (*p* < 0.05) higher expression. Moreover, the TCP group also had statistically significantly higher expression than the CS and GRADIENT groups at week 6. No significant differences were observed in ACAN expression over time in the PLGA group. The ACAN expression in the CS group at week 3 was statistically significantly higher than the ACAN expression at the other three time points in the group. The TCP group had statistically significantly higher ACAN expression at week 6 than at weeks 0, 1.5, and 3. Lastly, the GRADIENT group had statistically significantly higher ACAN expression at week 1.5 than ACAN expression at the other 3 weeks.

The COL1A1 (collagen I) expression (Figure 9D) of the GRADIENT at week 1.5 was 97-fold (*p* < 0.05) higher than the COL1A1 expression of the PLGA group, which was one of the only instances where a test group outperformed the PLGA control. In addition, the COL1A1 expression of the GRADIENT group was statistically significantly higher than the COL1A1 expression levels of the CS and TCP groups. No significant differences were observed in COL1A1 expression among groups at weeks 0, 3, and 6. Additionally, the week 1.5 COL1A1 expression of the GRADIENT group was statistically significantly higher than its COL1A1 expression at any other time point. No significant differences were observed over time in any other group in the COL1A1 expression.

#### RUNX2 and BGLAP

RUNX2 expression (Figure 9E) showed no significant differences among groups at week 0. However, at week 1.5, the PLGA and GRADIENT groups had statistically significantly higher RUNX2 expression than the expression levels of the CS and TCP groups, but were not significantly different from each other. At week 3, the PLGA group had statistically significantly higher RUNX2 expression than the other three groups. Moreover, the CS group at week 3 had significantly higher RUNX2 expression than the TCP group. Week 6 expression levels indicated that the TCP group had significantly higher RUNX2 expression than the CS and GRADIENT groups. The PLGA RUNX2 expression at week 3 was found to be statistically significantly higher than its corresponding values at week 0 and week 6, but was not significantly different from its week 1.5 value. The GRADIENT group RUNX2 expression at week 1.5 was statistically significantly higher than at its values at weeks 0 and 6, but did not differ significantly from its value at week 3. No significant differences over time were observed in the RUNX2 expression levels of the CS and TCP groups.

BGLAP expression (Figure 9F) showed no significant differences among groups at week 0 and no significant differences among the CS, TCP, and GRADIENT groups at week 1.5 (PLGA value at week 1.5 is not reported because of insufficient sample size). At week 3, the CS group had 10.4-fold (*p* < 0.05) higher BGLAP expression than the PLGA group. Moreover, the CS group had statistically significantly higher BGLAP expression than the TCP and GRADIENT groups. At week 6, the PLGA group had statistically significantly higher BGLAP expression than the GRADIENT group. In addition, the CS group expression level was significantly higher than the expression levels of the TCP and GRADIENT groups. The CS group BGLAP expression at week 3 was statistically significantly higher than its values at weeks 0, 1.5, and 6, respectively. In addition, the CS group BGLAP expression at week 6 was statistically significantly higher than its values at weeks 0 and 1.5, but was significantly lower than its week 3 value. No significant differences over time were observed in the BGLAP expression within any other group.

#### SPP1 and IBSP

The SPP1 (osteopontin) expression (Figure 9G) showed no significant differences among groups at week 0. At week 1.5, the GRADIENT scaffolds had 248-fold (*p* < 0.05) higher SPP1 expression than the PLGA group, another example of gene expression in a test group outperforming the PLGA control. Moreover, the SPP1 expression in the GRADIENT group was statistically significantly higher than the CS and TCP groups. No significant differences among the CS, TCP, and GRADIENT groups were observed in the SPP1 expression levels at week 3 (the PLGA group expression at week 3 is not reported due to insufficient sample size). Again, no significant differences among groups were observed at week 6. The CS group expression at week 3 was statistically significantly higher than at week 0. The GRADIENT group expression at week 1.5 was significantly higher than at weeks 0, 3, and 6. No significant differences over time were observed within any of the remaining two groups.

IBSP expression (Figure 9H) showed no significant differences among the CS, TCP, and GRADIENT groups at week 1.5 (the values for CS, TCP, and GRADIENT groups at week 0; and the PLGA group at week 1.5 are not reported because of insufficient sample size). At week 3, the IBSP expression of the CS group was 1.8-fold (*p* < 0.05) higher than the PLGA group. In addition, the CS group IBSP expression at week 3 was statistically significantly higher than the TCP and GRADIENT groups. The PLGA group at week 3 also had significantly higher expression than the TCP and GRADIENT groups. No significant differences among groups were observed at week 6. The CS group IBSP expression at week 3 was statistically significantly higher than at weeks 1.5 and 6. No significant differences were observed over time within any of the other groups.

## Discussion

The current study for the first time demonstrated the feasibility of raw material encapsulation in high molecular weight PLGA microsphere-based scaffolds that could potentially be used in large animal models or human patients. This work builds on our previous efforts that spoke of the advantages of raw material encapsulation (in conjunction with growth factors) toward creating a new tissue-specific ECM in low molecular weight PLGA scaffolds (Mohan et al., 2013). Furthermore, employing opposing gradients of CS and TCP to provide bioactive cues and building blocks for simultaneous chondrogenic and osteogenic differentiation of cells is a promising approach for osteochondral interfacial tissue engineering. Additionally, to the best of our knowledge, we are the first group to encapsulate TCP in microsphere-based scaffolds for the bone part of our scaffolds. Most of the other groups utilizing microsphere-based scaffolds have relied on other calcium phosphates and minerals for engineering the bone tissue (Cushnie et al., 2008; Lv et al., 2009; Tahriri and Moztarzadeh, 2014; Xu et al., 2014).

The scanning electron microscopy (SEM) images, depicting the overall porous nature of microsphere-based scaffolds with interconnections among the pores, were in agreement with our previous findings with these scaffolds fabricated with low molecular weight PLGA (Singh et al., 2008). Moreover, raw material encapsulation did not affect the spherical nature of the microspheres; however, it was found to have altered the microstructure of the microspheres. Specifically, the CS microspheres had a porous surface that could be attributed to the solvent removal process during the microsphere fabrication step, as we have also observed previously (Mohan et al., 2013). The presence of sub-micron pores on the CS microspheres contributed toward higher average porosity in these scaffolds compared to the other three groups. The TCP encapsulating microspheres, on the other hand, did not possess pores on their surfaces, but had a rough surface instead. The surface roughness of these microspheres, specifically the presence of ridge-like features, may have resulted from the partitioning of TCP particles on the surface of the microspheres. These surface characteristics of raw material encapsulating microspheres may have great implications in cell attachment or anchorage and also in diffusion of nutrients and wastes in and out of the scaffolds (Persson et al., 2014; Wu et al., 2014). Furthermore, raw material encapsulation impacted the cellular morphology of the seeded rBMSCs. Flat cells with significant cell spreading were observed in the CS and GRADIENT groups while cluster forming round cells could be seen in the TCP and GRAIDENT groups. Though the GRADIENT group contained both flat and round cells, no differences in cell morphologies were observed in cells from distinct regions of the scaffold. The different cell morphologies on microsphere-based scaffolds might suggest that cells responded favorably to the encapsulated raw materials, at least initially, which may have influenced their differentiation along discrete pathways. This initial cellular response to encapsulated raw materials could have pivotal significance in regenerating interfacial tissues that require differentiation of cells from a single source along multiple pathways.

Mechanical testing results demonstrated the compressive moduli of microsphere-based scaffolds to be in the range of articular cartilage (0.1–0.9 MPa) and within an order of magnitude of the moduli for cancellous bone (0.01–2 GPa) (Keaveny and Hayes, 1993; Mansour, 2003; Williams et al., 2003). Moreover, the elastic modulus of TCP scaffolds at week 0 was found to be at least three times as large as any other group, thereby conforming to the observations of Lv et al. demonstrating that calcium phosphates enhance the mechanical properties of polymeric scaffolds (Lv et al., 2009). However, at week 6, the cell seeded TCP constructs had significantly higher modulus than the CS group alone. All the other groups, except for the CS group, had an increase in their elastic moduli from week 0. Differences among groups in degradation rates of the scaffolds, cell proliferation within the scaffolds, and ECM deposition could have all contributed to the increase in moduli. PLGA microspheres are known to degrade via bulk erosion where the rate-limiting step is the diffusion of water molecules into the microsphere core. CS microspheres because of their porous nature may have allowed faster diffusion of the water molecules into their core, thereby initiating the polymer degradation more quickly than in the other three groups. Higher glycolic acid content in PLGA (PLGA50:50) of CS microspheres may have further accelerated polymer degradation in the CS group (Alexis, 2004). Additionally, swelling (Table S3 in Supplementary Material) caused by penetration of water inside of the microspheres may have also played a part in the drop in elastic modulus of CS scaffolds (Mohan et al., 2013). On the other hand, swelling was absent (PLGA and TCP groups) or less pronounced (GRADIENT group) in the other three groups compared to the CS group, which may have prevented the drop in elastic moduli of scaffolds from the PLGA, TCP, and GRADIENT groups at week 6. Moreover, polymer composition (PLGA75:25) and microsphere morphology (absence of minute pores on surface) may have allowed the PLGA, TCP, and GRADIENT scaffolds to further retain their mechanical properties. Surprisingly, the PLGA scaffolds had a tremendous increase in modulus from week 0 to week 6, translating to an elastic modulus orders of magnitude higher than the moduli of the other three groups at week 6. We previously observed a similar trend in elastic moduli in raw material encapsulating low molecular weight PLGA scaffolds where deviations from the overall scaffold structure at week 6 led to a significant increase in elastic modulus (Mohan et al., 2013). Additionally, the elastic moduli of high molecular weight PLGA acellular scaffolds at week 6 (unpublished data) also hinted toward a similar phenomenon. Therefore, it is speculated that cellular contributions, in conjunction with polymer degradation, led to microscopic changes in the scaffold morphology (closure of pores) that caused the elastic moduli of PLGA constructs to jump at week 6. However, further investigation is needed to better understand the degradation in these high molecular weight PLGA scaffolds and the mechanism of increase in their compressive moduli with time. Altogether, results from the mechanical testing provided information that would be valuable in designing microsphere-based scaffolds for future *in vitro* and *in vivo* studies in rabbits, sheep, etc.

The biochemical content results were found to be consistent with the SEM observations. A small number of cells were observed on the PLGA scaffolds at Day 10, which agreed with the DNA content analysis that revealed low quantities of DNA on these scaffolds throughout the 6-week culture period. By contrast, the DNA contents of all the raw material encapsulating groups increased over time with significant differences appearing at week 6. Our DNA results on microsphere-based scaffolds suggest that raw material encapsulation encouraged rBMSC proliferation on these scaffolds, thus agreeing with the findings of some other groups showing that the raw materials such as CS and β-TCP could cast a positive influence on the proliferative capacity of rBMSCs (Takahashi et al., 2005; Uygun et al., 2009; Todo and Arahira, 2013; Kim et al., 2014). GAG data showed that the CS group had at least a threefold higher GAG content than the rest of the groups at week 6. Since the GAG content of acellular constructs (Table S1 in Supplementary Material) was subtracted at each time point, it is to be stressed that the data primarily represented GAG secreted by the cells and also released CS entrapped within the newly synthesized ECM. A trend similar to GAG content was seen in the HYP content of CS scaffolds suggesting that the encapsulated CS played a significant role in enhancing the cellular GAG and collagen secretion, thus having a modulatory effect on the seeded rBMSCs. However, observance of lower normalized GAG and HYP content in the CS group than the PLGA group suggest that the bioactive effects seen due to CS encapsulation may have been primarily due to the improvement in cellularity without sacrificing biosynthesis on a per cell basis. Calcium content analysis revealed some unanticipated results. The CS group had significantly higher net calcium content than the other groups at week 6, and the PLGA group was higher in calcium per DNA content compared to rest of the groups at that time. The counter-intuitive phenomenon of high calcium (or calcium per DNA) contents in the CS and PLGA groups could be attributed to culture medium components, such as DEX, β-GP, and IGF-I. DEX is a glucocorticoid, which is used extensively *in vitro* as an osteogenic factor. β-GP is the common source for MSCs to form CaP deposits *in vitro* (Shi et al., 2010; Fiorentini et al., 2011). IGF-I is an anabolic signal that does not necessarily influence the proliferation and differentiation of MSCs toward osteoblasts on its own, but it is an important molecule directing the differentiation of already osteogenically committed cells (Hayrapetyan et al., 2014). Thus, the presence of these components likely influenced the commitment of rBMSCs on the microsphere-based scaffolds toward osteogenesis. Furthermore, the ALP activities of the TCP and GRADIENT groups at week 0 were higher than their activities at week 6. The elevated ALP activities in these constructs at earlier time points may have been due to the medium components. However, failure to observe a similar effect in the other two groups hint that TCP encapsulation might have influenced their behavior initially as seen with the SEM micrographs as well. Lastly, higher normalized HYP and calcium contents in the TCP encapsulating scaffold groups than the CS group at later time points suggest that TCP encapsulation may have improved rBMSC performance by promoting their differentiation in addition to enhancing their proliferation (as seen with DNA content results).

Gene expression results were in agreement with the other results of the study. Relatively higher expressions of SOX9, COL2A1, and RUNX2 by the cells in the PLGA group at week 3 followed by higher mineral content at week 6 (as indicated by the biochemical data) suggest that the DEX in culture the medium may have caused the rBMSCs in the PLGA scaffolds to go down the osteogenic pathway via a cartilage-like intermediate. Higher expression levels of chondrogenic markers (collagen II and aggrecan), in conjunction with up regulation of osteogenic markers (BGLAP and IBSP) by the cells in the CS group at week 3 than compared to the initial time points, suggest a similar phenomenon as observed in the PLGA group. Lower expression of BGLAP and IBSP in the TCP group than the CS group suggests that TCP presence inhibited expression of osteogenic markers by creating a substrate environment that was already high in mineral content, a phenomenon previously observed with hydroxyapatite encapsulating low molecular weight PLGA microsphere-based scaffolds (Dormer et al., 2012a). The cells in the GRADIENT group showed relatively higher expression of ACAN and SPP1 (along with higher expression of RUNX2 by the cells in the group than at week 0) than the cells in the PLGA control group at week 1.5. The higher expression of some chondrogenic and osteogenic markers in the GRADIENT group at earlier time points may be due to faster maturation of rBMSCs toward cartilage- and bone-like cells in this group; however, more evidence is needed to reinforce this speculation.

Overall, the results of the current study indicate that raw material encapsulation into microsphere-based scaffolds influenced the behavior of the seeded rBMSCs. Differences in the cell morphologies and greater cell numbers in the raw material groups leading to enhanced matrix synthesis in these groups demonstrates that the raw materials provide a head start in the (re)generation of tissues. It is of interest to infer the amount of matrix synthesized by cells in the scaffolds beyond the exogenously included amounts. Therefore, the biochemical content (CS and calcium) for the acellular constructs was subtracted from the content of the cell seeded constructs, assuming that the acellular scaffolds degrade and release encapsulated molecules at the same rate as their cellular counterparts. However, we acknowledge that this assumption is weak as cells synthesizing new matrix, and perhaps altering the surrounding pH, etc., will influence the polymer degradation rate, but with the higher molecular weight PLGA, it should be a reasonable approximation that allows us to better evaluate differences among groups due to cellular contribution. In addition, static seeding approach employed in this study has limitations associated with it due to the manual- and operator-dependent nature of the process. However, we followed a uniform manual seeding procedure, and we think that the differences observed in the DNA content at week 0 (24 h post seeding) among various scaffold type might have resulted more from the differences in cell attachment arising due to differences in scaffold composition than arising from variations in cell seeding. Moreover, we did not specifically explore the dosing effect of CS and TCP, but our group has demonstrated in the past that the concentration of the raw materials can have a significant effect on the differentiation of the cells (Dormer et al., 2012a). Additionally, higher cell number, greater biochemical content, and relatively higher expression of some osteogenic and chondrogenic markers in the GRADIENT group accentuated the advantages of using gradient-based strategies for engineering the osteochondral interface. However, we recognize that these scaffolds not being amenable to histology due to the stiffness of the polymer constructs, given the high molecular weight and slow degradation of the PLGA, was a limitation of the study that would have further elaborated the differences among groups based on their regional material composition, but we have substantiated previously both *in vitro* and *in vivo* that regionalized tissue formation occurs in raw material gradient microsphere-based engineered constructs (Mohan et al., 2011, 2013). Furthermore, the initial effects of raw material encapsulation on a per-cell basis might have been obscured by the culture medium components that appeared to favor osteogenesis. However, it is to be noted that *in vitro* advancements observed initially with raw material encapsulation could translate *in vivo* to a more favorable interaction with infiltrating MSCs, and perhaps facilitate differentiation in a native environment rather than in a medium-governed environment. Lastly, an important consideration in designing scaffolds for clinical use is determining the mechanical integrity. We have shown in our prior work that microsphere-based scaffolds possess adequate mechanical properties for the regeneration of osteochondral tissues and the encapsulation of raw materials may impact those properties (Dormer et al., 2010, 2012a; Mohan et al., 2013). Our mechanical testing results in the current study also agreed with our previous findings; additionally, the results also suggested that the mechanical properties of microsphere-based scaffolds can be impacted by scaffold degradation and cellular matrix synthesized by the seeded cells. Additional cyclic testing in the future may yield interesting information about degree of hysteresis and narrower strain ranges about a fixed strain point (e.g., 5%), with a frequency sweep could yield interesting tan delta profiles as well, which we will consider for future studies.

Altogether, the overall findings emphasize the need to further refine the technology, perhaps by adjusting raw material concentration or by altering PLGA degradation rate. The degradation of the polymer will play a key role in tissue regeneration *in vivo*, where premature failure in scaffold mechanical properties can have a deleterious effect on the regenerating tissue, and extended degradation by contrast could become an obstacle to tissue regeneration. Therefore, it is important to identify a polymer with a biodegradation rate comparable to the neo-tissue formation rate. Additionally, identifying raw material concentrations that are most efficacious in promoting osteogenesis and chondrogenesis would yield valuable information, which could then be leveraged for tailoring scaffold degradation in future sheep or any other large animal model studies. Nevertheless, the current study highlights several benefits of raw material microsphere gradient scaffold technology. The raw material encapsulating microsphere-based scaffolds attempt to regenerate both cartilage and bone simultaneously, thus stressing on the importance of growing cartilage and bone within the physical proximity of each other; many signaling pathways and endogenous proteins responsible for progenitor cell commitment to the osteoblast or chondrocyte lineages have a high degree of interrelatedness (Gordeladze et al., 2009). The raw materials apart from being conductive to tissue (re)generation can also provide inductive signals to the surrounding cells guiding their differentiation. In addition, the raw materials provide clinical significance to microsphere gradient scaffolds, as these scaffolds may be tactically placed for swifter and less costly regulatory approval.

## Conclusion

The present study assessed the *in vitro* response of microsphere-based scaffolds with clinical relevance fabricated using a raw material approach. Overall, the results demonstrated that the primary improvements observed with the raw materials-CS and TCP were more due to greater initial interaction with cells and greater cellularity with comparable performance on a per-cell basis rather than on specifically driving differentiation. Moreover, the medium-governed environment that seemed to favor osteogenesis concealed the initial *in vitro* advancements observed with raw material encapsulation. Additionally, there was also evidence of faster maturation of rBMSCs in the raw material GRADIENT constructs that can be leveraged further to engineer the complex osteochondral interface. Therefore, a strategy combining the “building block” side of the raw material philosophy (as we have done here) with the “signaling” side, for example, by including hydroxyapatite with the TCP, or maybe TGF-β with CS, or by altering the dose of CS (without TGF-β); in a scaffold with a biodegradation rate comparable to the neo-tissue formation rate, we may be able to achieve the differentiation profiles we seek *in vitro*.

## Conflict of Interest Statement

The authors declare that the research was conducted in the absence of any commercial or financial relationships that could be construed as a potential conflict of interest.

## Supplementary Material

The Supplementary Material for this article can be found online at http://journal.frontiersin.org/article/10.3389/fbioe.2015.00096
