# Supplementary material for: Microsphere-Based Scaffolds Carrying Opposing Gradients of Chondroitin Sulfate and Tricalcium Phosphate
Source: Front Bioeng Biotechnol. 2015 Jul 1;3:96. doi: 10.3389/fbioe.2015.00096 (PMC4486839; doi:10.3389/fbioe.2015.00096)
Supplement: Supplementary file 1 [file Data_Sheet_1.DOCX]

***Supplementary Material***

**Microsphere-Based Scaffolds Carrying Opposing Gradients of**

**Chondroitin Sulfate and Tricalcium Phosphate**

**Vineet Gupta^1^, Neethu Mohan^2^, Cory J. Berkland^1, 3^, and Michael S. Detamore^1, 4^***

^1^Bioengineering Graduate Program, University of Kansas, Lawrence, Kansas, USA

^2^Division of Tissue Engineering and Regeneration Technologies, Biomedical Technology Wing, Sree Chitra Tirunal Institute for Medical Sciences and Technology, Trivandrum, Kerala, India

^3^Department of Pharmaceutical Chemistry, University of Kansas, Lawrence, Kansas, USA

^4^Department of Chemical and Petroleum Engineering, University of Kansas, Lawrence, Kansas, USA

*** Correspondence:** Michael S. Detamore, Ph.D.**,** Professor**,** Department of Chemical and Petroleum Engineering, The University of Kansas, 4149 Learned Hall, 1530 W. 15th Street, Lawrence, KS 66045-7618, USA.

Email: [detamore@ku.edu](mailto:detamore@ku.edu)

1. **Supplementary Figures and Tables**

## Supplementary Tables

**Supplementary Table 1:** GAG content measured over time in acellular scaffolds from the CS and GRADIENT groups. All values are expressed as the average ± standard deviation (n = 3).

| **Group** | **GAG (μg/scaffold)** | | |
| --- | --- | --- | --- |
|  | **Week 0** | **Week 3** | **Week 6** |
| CS | 17.4 ± 9.4 | 15.9 ± 5.0 | 16.5 ± 3.8 |
| GRADIENT | 18.4 ± 23.9 | 9.3 ± 11.3 | 4.4 ± 2.5 |

**Supplementary Table 2:** Calcium content measured over time in acellular scaffolds from the TCP and GRADIENT groups. All values are expressed as the average ± standard deviation (n = 3).

| **Group** | **Calcium (μg/scaffold)** | | |
| --- | --- | --- | --- |
|  | **Week 0** | **Week 3** | **Week 6** |
| TCP | 13.8 ± 19.3 | 3.3 ± 0.9 | 2.7 ± 2.5 |
| GRADIENT | 7.7 ± 3.7 | 6.9 ± 1.3 | 17.7 ± 6.5 |

**Supplementary Table 3:** The dimensions of the constructs used for mechanical testing. All values are expressed as the average ± standard deviation (n = 3-5), p < 0.05 *statistically significant difference from its Week 0 value and $statistically significant difference from PLGA group at that time point.

| **Group** | **Week 0 (Acellular)** | | **Week 6 (Cellular)** | |
| --- | --- | --- | --- | --- |
|  | **Diameter (mm)** | **Height (mm)** | **Diameter (mm)** | **Height (mm)** |
| PLGA | 4 ± 0 | 6.5 ± 0.7 | 4 ± 0 | 6.8 ± 0.3 |
| CS | 3.9 ± 0.1^$^ | 6.3 ± 0.9 | 5.7 ± 0.1^*$^ | 10 ± 1^*$^ |
| TCP | 4.1 ± 0.2 | 6.4 ± 0.2 | 3.8 ± 0.1^*^ | 6.3 ± 1.0 |
| GRADIENT | 4.1 ± 0.2 | 6.8 ± 0.2 | 4.4 ± 0.2^*$^ | 7.6 ± 0.5 |

## Supplementary Figures


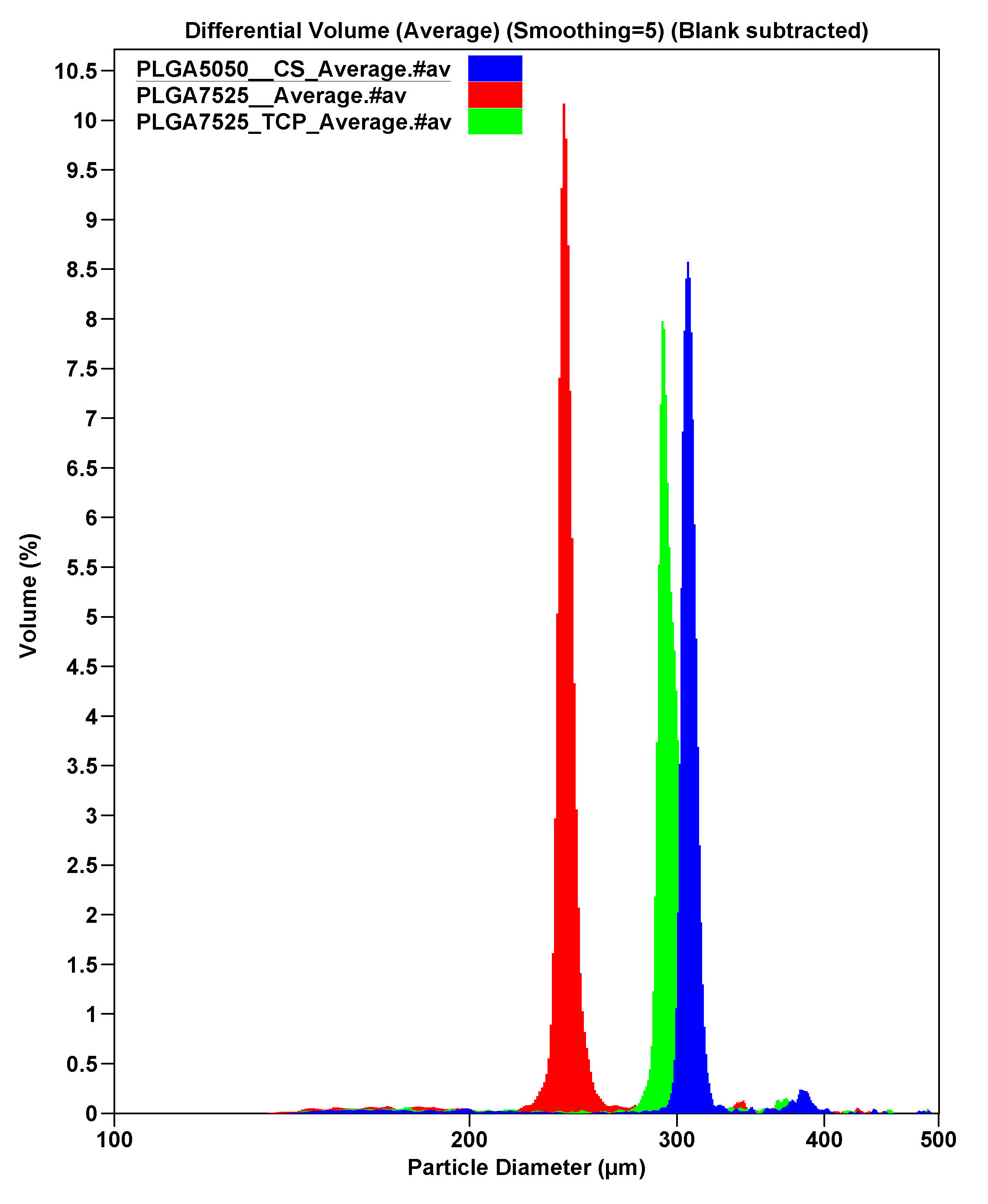


**Supplementary Figure 1. Microsphere size distribution graph.** Mean particle size for PLGA, CS, and TCP groups were 238 μm, 296 μm, and 283 μm, respectively.
